# A Short Course of Celecoxib Prevents Heterotopic Ossification Following Cementless Total Hip Arthroplasty

**DOI:** 10.3390/life13040944

**Published:** 2023-04-04

**Authors:** Hamid Al Badi, Michael Tanzer, Anas Nooh, Brandon Hall, Adam Hart

**Affiliations:** Jo Miller Orthopaedic Research Laboratory, Division of Orthopaedic Surgery, McGill University, Montreal, QC H3G 1A4, Canada

**Keywords:** heterotopic ossification, prevention, Celecoxib, hip, arthroplasty, cementless, prophylaxis, Brooker, COX-2, NSAID

## Abstract

Heterotopic ossification (HO) is a common complication after total hip arthroplasty (THA) and can result in pain and loss of motion of the hip. This is the first study in the literature to determine if a short course of Celecoxib is effective in the prevention of HO in patients undergoing cementless THA. In this retrospective study of prospectively collected data, consecutive patients undergoing a primary cementless THA were reviewed at a 2-year follow-up. The Control group consisted of 104 hips that did not receive Celecoxib (Control group), while the 208 hips in the Celecoxib group received 100 mg twice daily for 10 days. Radiographs, patient-recorded outcome measures and range of motion (ROM) were evaluated. Overall, there was a significantly decreased incidence of HO in the Celecoxib group (18.7%) than in the Control group (31.7%) (*p* = 0.01). The odds that a patient developed HO using Celecoxib were 0.4965 times the odds that a patient developed HO without treatment. Clinically, the Celecoxib group demonstrated significantly greater improvement in their mean WOMAC stiffness (0.35 vs. 0.17, *p* = 0.02) and physical function scores (3.26 vs. 1.83, *p* = 0.03) compared to those in the Control group, but there was no difference in the ROM between the two groups. This study is the first to demonstrate that the lowest dose of Celecoxib for a short course of only 10 days is a simple and effective prophylactic treatment option that can significantly reduce the incidence of HO following cementless THA.

## 1. Introduction

First described by Reidel in 1883 [1], heterotopic ossification (HO) is defined as the pathological formation of mature lamellar bone [2] in muscle and soft tissue where physiologically there is no osseous tissue present [3]. Many predisposing factors including neurological injury to the spinal cord or brain, major joint surgery and burns have been associated with HO [4]. HO is a common complication after total hip arthroplasty (THA), with an incidence ranging from 8% to 63% [4,5,6,7,8]. Many risk factors have been shown to be associated with an increased incidence of HO following THA, including male gender, older age, hypertrophic osteoarthritis, Paget’s disease, post-traumatic osteoarthritis, osteonecrosis, rheumatoid arthritis, diffuse idiopathic skeletal hyperostosis, ankylosing spondylitis, a previous history of HO and revision THA [7,9,10,11]. Patients with a previous history of HO in an ipsilateral joint are at a greater risk of developing HO than those in whom the previous HO was on the contralateral side [12]. Other risk factors related to the surgical technique include the extent of soft tissue dissection, bone trauma, the persistence of bone debris (reaming and marrow) and the presence of a hematoma [5]. Ashton et al. showed that the posterior approach to the hip was associated with a lower rate of HO than the anterolateral or transtrochanteric approaches [12].

Although the exact etiology of HO remains unclear, it can be categorized as traumatic, neurological or genetic. HO following hip arthroplasty surgery is considered traumatic in nature, with the abductor muscles being most commonly involved [5]. While the pathogenesis of HO formation following THA is still unknown, different hypotheses have been proposed. Friedenstein suggested that migrated cells from bone marrow stimulate ectopic osteogenesis in connective tissue [13], while Ackreman [14] suggested that muscle lesions or interstitial hemorrhagic foci lead to muscle degeneration, the proliferation of perivascular connective tissue and finally to bone metaplasia. Urist and McLaren hypothesized that a lesion of the periosteum causes the differentiation of the osteogenetic cells and the occurrence of periarticular bone formation [15].

Clinical manifestations of HO can vary from being an asymptomatic incidental radiological finding to reduced range of motion (ROM) and complete ankylosis [5,16,17,18]. Significant functional limitations from HO due to decreased ROM and pain occur in 3% to 10% of patients after THA [9,19,20,21]. Limitations in range of motion may compromise sitting, dressing, transfers and even ambulation [22]. Therefore, the prevention of such complications is important to optimize patients’ functional outcomes after THA. 

The increased bone turnover that occurs with HO after THA can be detected by blood tests measuring specific osteoblastic and osteoclastic markers (CTX-1 and P1NP), as early as one week post-operatively [23]. In hips that form HO, a bone scan will demonstrate increased uptake as early as three weeks after the surgery, while plain radiographs take up to four to six weeks to show any radiographical changes [18]. Extensive bone formation can occur within three months, but the full maturation of heterotopic bone takes up to one year after hip arthroplasty [1]. Therefore, in the cases of severe HO causing impairment of motion, surgical excision needs to be delayed for at least one year following THA [24].

Different methods have been described to prevent HO following THA, including radiotherapy and NSAIDs [5]. The mechanism of action of ionizing radiation therapy is due to the inhibition of the fast-dividing osteoprogenitor cells that are present within the first week and which then differentiate into mature cell types, such as osteoblasts [5,21,25,26,27,28,29,30]. A recent meta-analysis has indicated that radiotherapy is the most effective prophylactic option for preventing HO after THA [31] but can be associated with impaired surgical wound healing, the inhibition of bone growth into cementless arthroplasties and the prevention of osteotomy healing [5,11,22,27,32]. Non-steroidal anti-inflammatory drugs (NSAIDs) are effective and widely used for HO prophylaxis following THA [5]. NSAIDs work by inhibiting the activity of the enzyme cyclooxygenase (COX), and its two isoforms, COX-1 and COX-2. NSAIDs work by inhibiting the activity of cyclooxygenase (COX) enzymes. COX enzymes are utilized by the cell for the synthesis of prostaglandins involved in inflammation and thromboxane, which is involved in blood clotting. NSAIDs prevent HO formation by inhibiting the formation of prostaglandin E2, as well as by inhibiting pre-osteoblast differentiation, bone formation and resorption [33,34,35]. However, although the prolonged use of NSAIDs can prevent HO, they are associated with an increased risk of gastrointestinal (GI) and cardiovascular side effects [15]. A selective COX-2 inhibitor, such as Celecoxib, has the advantage of minimizing GI side effects, can be taken with postoperative anticoagulation and has become an effective modality in Enhanced Recovery After Surgery (ERAS) programs to minimize opioid use and to facilitate early discharge [15,36]. A network meta-analysis of 31 randomized clinical trials comparing strategies for preventing heterotopic ossification after THA found no significant difference between selective NSAIDs and non-selective NSAIDs in terms of the overall incidence of HO [31]. However, side effects can still occur with a selective COX-2 inhibitor, but these can be mitigated by decreasing the duration of use [37,38,39]. Therefore, it is important to understand if a short course of a COX-2 inhibitor is effective in preventing HO after THA. 

To date, no studies have evaluated the formation of HO after THA with and without the use of a low dose of Celecoxib for a short duration with two years of follow-up. The aims of this study were to investigate whether a short course of Celecoxib is effective in the prevention of HO in patients undergoing cementless THA and to determine if this would result in any difference in postoperative ROM and function.

## 2. Materials and Methods

Institutional review board approval was obtained prior to the onset of the study. In this retrospective study of prospectively collected data, 312 consecutive hips that underwent a primary cementless THA prior to and after the introduction of Celecoxib into our ERAS pathway were reviewed. All operations were performed by a single surgeon using the same cementless highly porous implants—Trilock BPS stem and a Pinnacle cup (DePuy Synthes, Warsaw, IN, USA). All surgeries were performed through a mini posterior approach. After splitting the gluteus maximus, the gluteus medius and minimus were retracted, and the external rotators were tagged with a suture and detached from the greater trochanter utilizing electrocautery. The posterior capsule was then opened in a T-shaped fashion. After implantation, the wounds were copiously irrigated with saline, the capsule was closed in a running fashion and the external rotators were reattached to the greater trochanter with sutures. All patients were allowed to fully bear weight on their arthroplasty immediately after the surgery. The length of follow-up was 2 years for all THAs. 

The THAs were divided into two groups depending on whether or not they received Celecoxib postoperatively. The Control group had no exposure to Celecoxib and included THAs performed prior to January 2013. The ERAS pathway included only Acetaminophen and Hydromorphone for pain relief. After January 2013, Celecoxib was added to the ERAS pathway to minimize pain and opioid use, and these THAs formed the Celecoxib group. No other NSAIDs were included in the ERAS pathway. The Celecoxib group received Celecoxib 100 mg orally twice daily for 10 days postoperatively. Since the Celecoxib group was over a longer period of time, the number of hips in the group was chosen to be twice that of the Control group. No patients in either group had established risk factors for HO preoperatively. No patients in either group received extended prophylaxis with any NSAID or perioperative radiotherapy. Any patients taking other NSAIDs postoperatively for any reason and those with inflammatory arthritis were excluded from the study. 

A total of 104 THAs (96 patients) were in the Control group and 208 THAs (187 patients) were in the Celecoxib group. There was no significant difference in the demographics between the two groups, with the patients being primarily males in their mid-sixties and having a diagnosis of osteoarthritis (Table 1 and Table 2).

A single-blinded observer evaluated the pre-operative and at two-year post-operative anteroposterior (AP) radiograph of the pelvis and AP and lateral radiographs of the involved hip for the presence of HO. The HO was graded according to the Brooker classification system: Class I, islands of bone within the soft tissue about the hip; Class II, bone spurs from the pelvis or proximal end of the femur, leaving at least one centimeter (cm) between opposing bone surfaces; Class III, bone spurs from the pelvis or proximal end of the femur, reducing the space between opposing bone surfaces to less than 1 cm; and Class IV, apparent bone ankylosis of the hip [40]. 

All patients were evaluated clinically preoperatively and then at two years postoperatively. Clinical evaluation included the range of motion (ROM) of the hip, as well as patient-recorded outcome measures (PROMs). PROMs included the Harris hip score (HHS), the Western Ontario and McMaster Universities Osteoarthritis Index (WOMAC) for pain, stiffness, and physical function, the 12-item Short Form Health Survey (SF-12) physical and mental components and the University of California Los Angeles (UCLA) activity scale. The HHS is a ten-item score measuring four domains that were developed for the assessment of the results of hip replacement surgery. The first domain is pain, evaluated in terms of severity, its effect on activities and the need for pain medications. The second part is function, involving daily activities (stair use, using public transportation, sitting and managing shoes and socks) and gait (limp, support needed and walking distance). The third domain is deformity, measuring hip flexion, adduction, internal rotation and leg length discrepancy. The last domain is the range of motion measuring hip flexion, abduction, adduction and internal and external rotation. The score has a maximum of 100 points (best possible outcome) [41,42]. The 12-item Short Form Health Survey (SF-12) physical and mental components is a health-related quality-of-life questionnaire measuring eight health domains to assess physical and mental health [43]. The WOMAC is a self-administered questionnaire that is composed of 24 questions categorized into three subscales (pain, stiffness and physical function). Each question is graded from 0 to 4, with 0 meaning none and 4 meaning extremely [44]. The UCLA scale is a scale ranging from one to ten indicating the patient’s level of activity. Grade 1 is defined as “no physical activity, dependent on others” and 10 means “regular participation in impact sports” [45]. Patient demographics, including age, sex and diagnosis were obtained from a chart review. 

All statistical analyses were conducted using SPSS Version 21 (IBM Corp, Armonk, NY, USA). Descriptive analyses were used for patients’ demographics and radiographic characteristics. A paired T-test was conducted to determine the significance between HHS, WOMAC, SF-12 and UCLA activity scores in the Control and Celebrex groups. A Chi-square test was utilized to analyze the difference in Brooker class bone formation between the two groups. A *p*-value <0.05 was considered statistically significant.

## 3. Results

Overall, there was a significantly decreased incidence of HO in the Celecoxib group (18.7%) compared to that in the Control group (31.7%) (*p* = 0.01) (Table 3). 

The odds that a patient developed HO using Celecoxib were 0.4965 times the odds that a patient developed HO without treatment. Mild HO (Brooker I and Brooker II) was more common in the Control group (27.9%) than in the Celecoxib group (15.8%) (*p* = 0.01). There was no difference in the number of hips that formed Brooker II and Brooker III HO in the Control group (6.7%) compared to that in the Celecoxib group (7.2%) (*p* = 0.88). Severe HO (Brooker III and Brooker IV) occurred in 3.8% of the hips in the Control group and 2.9% of the hips in the Celecoxib group (*p* = 0.64). No patients in either group developed Brooker class IV HO. 

Clinically, the Celecoxib group demonstrated significantly greater improvements in their mean WOMAC stiffness (0.35 vs. 0.17, *p* = 0.02) and physical function scores (3.26 vs. 1.83, *p* = 0.03) compared to those in the Control group (Table 4). 

There was no significant difference between the Control and Celecoxib groups with respect to their WOMAC pain score, HHS, UCLA Activity Score, and their SF-12 score (Table 4). In addition, there was no difference in the ROM between the two groups, with both groups demonstrating normal hip motion after their hip arthroplasty (Table 5).

## 4. Discussion

Preventing the formation of HO after THA is advisable since this common complication can negatively impact a patient’s outcome. The extensive formation of ectopic bone can be painful, significantly reduce the range of motion and function of the hip joint and necessitate a complex surgery to remove the ossified tissue [15]. This study demonstrated that a short course of a COX-2 inhibitor, Celecoxib, significantly reduced the formation of HO after cementless THA. With only 10 days of treatment with Celecoxib, the odds that a hip developed HO postoperatively was lowered by 50.1%. Although the short-term use of Celecoxib had no effect on the range of motion of the hips, the Celecoxib group demonstrated significantly greater improvement in their mean WOMAC stiffness and physical function scores. 

While radiotherapy and NSAIDs are effective prophylactic options for the prevention of postoperative HO [21,27,46,47,48], radiotherapy requires specialized equipment and expertise that is not available in all centers and can result in significant complications [49,50,51]. As a result, postoperative NSAIDs are the most common HO prophylactic option, with Indomethacin being the historical gold standard [33,52,53,54,55]. However, non-selective NSAIDs, such as Indomethacin, interact with postoperative anticoagulation and are associated with the premature discontinuation of therapy due to gastrointestinal complications [38,39,56]. Nonselective NSAIDs have 1.85 times the odds of having gastrointestinal side effects and 3.2 times the odds of having any complication compared to those when using selective NSAIDs to prevent HO after THA [31]. While COX-2 selective inhibitors cause fewer GI side effects, there has been controversy regarding their effect on the cardiovascular system [39,57,58,59,60,61]. However, the cardiovascular risk occurred after a long period of use of at least 12 months [57,58]. Selective NSAIDs have been shown to have a significantly lower incidence of non-gastrointestinal side effects (odds ratio = 0.43) than nonselective NSAIDs when used for HO prophylaxis [31]. In addition, Celecoxib has become an integral part of post-operative medication to control pain and reduce the need for narcotics in ERAS pathways [36].

Previous studies on HO prophylaxis after THA with the selective NSAID Celecoxib have demonstrated the same efficacy in preventing HO following THA as that of Indomethacin [15,62,63]. A recent meta-analysis of 31 randomized clinical trials found no significant difference in the overall incidence of HO between selective NSAIDs and non-selective NSAIDs in the prevention of HO after THA [31]. Compared to controls, both nonselective NSAIDs and selective NSAIDs had a significantly lower incidence of Brooker II and III HO. Studies looking specifically at Celecoxib to prevent HO following THA have demonstrated its effectiveness, but with a longer duration of use compared to that in this study [6,15,22,64,65]. Romano and Zhao reported that a 20-day or 6-week course of Celecoxib was as effective as Indomethacin in preventing HO [15,65]. 

Only three studies have evaluated the effectiveness of postoperative Celecoxib, compared to a control group with no treatment, in preventing HO after THA [6,22,66]. Lavernia et al. [6] found that HO after THA, through a posterior approach, was significantly reduced in 72 hips that received Celecoxib 200 mg BID for 4 weeks, compared to that in 98 hips that did not. Despite doubling the dose and increasing the duration of treatment by 2.8 times compared to those in this study, the number of cases with Brooker II, III or IV was not different from our results (6.9% vs. 7.2%). Naylor et al. also demonstrated that Celecoxib 200 mg twice daily was effective in preventing HO after THA [66]. Patients who did not receive Celecoxib had a 14.3% rate of HO, versus 4.3% in the Celecoxib-treated group. Their incidence of severe HO (Brooker III or IV) was 2.2% in the control group and 0.3% in the Celecoxib group. Unlike in the present study, the patients received twice the dose of Celecoxib for 3 weeks and the surgery was performed through a direct anterior approach. The US Food and Drug Administration (FDA) guidelines for Celecoxib dosage in osteoarthritis is 200 mg per day administered as a single dose or as 100 mg twice daily [67]. Furthermore, they recommend using the lowest effective dose for the shortest duration possible to minimize the potential risk for an adverse cardiovascular event and to minimize gastrointestinal risks. In the only previous study comparing Celecoxib 200 mg daily to a control group with no treatment, Celecoxib was given for 6 weeks postoperatively [22]. After 6 weeks of treatment following THA through a posterior approach, 13% of the Celecoxib-treated hips developed HO, compared to 32% in the control group. These findings are similar to this study where 19% of the Celecoxib-treated hips and 32% of the controls developed HO with only 10 days of treatment. However, the incidence of severe HO (Brooker III or IV) was 10.4% in the study by Oni et al. with 6 weeks of treatment, compared to 2.9% in this study with 10 days of Celecoxib. The findings in this study are in agreement with the conclusion of the systematic review by Haffer et al. that 9 days of NSAID is the minimum duration of treatment time needed to prevent HO after THAs and minimize side effects, such as GI symptoms [56]. 

This study showed similar findings to the trial performed by Saudan et al. that compared the non-selective COX inhibitor Ibuprofen with the selective COX-2 inhibitor Celecoxib. Total hip replacement patients were randomized to either Celecoxib 200 mg bid or Ibuprofen 400 mg tid for ten days after surgery. They demonstrated that HO was more common in the Ibuprofen group with twice the dose used in this study, and Brooker HO was classified as none in 59.0%, Brooker class I in 35.9% and class II and III in 5.1% [64]. In comparison, in this study, Celecoxib prevented HO formation in 81% of the patients compared to 68% in the control group. Unlike in the present study, Saudan et al. only followed their patients for three months even though it is recognized that HO can progress for up to one year after surgery [24]. No other studies have compared a short course of Celecoxib to a short course of a non-selective NSAID. However, a short course of 14 days or less of non-selective NSAIDs has also been shown to be effective in preventing HO after THA. In a randomized trial of 41 hips, Kjaersgaard-Andersen et al. compared Indomethacin 25 mg three times daily to the placebo [55]. At the 3-month follow-up, none of the hips in the Indomethacin group developed significant HO, and the overall findings favored Indomethacin prophylaxis. A randomized control trial comparing a 14-day treatment with Ibuprofen (1200 mg daily) or placebo found that the risk of developing ectopic bone of any grade or severe HO was significantly decreased [68]. However, as a result of the significantly increased risk of major bleeding complications in the Ibuprofen group, the authors recommended against the use of this non-selective NSAID for HO prophylaxis after THA.

While our study had several strengths, it has some limitations. Firstly, it had a nonrandomized design with a selection bias intrinsic to the retrospective nature of this study. The effects of this limitation were minimized by using a study population comprised of consecutive THA cases before and after January 2010. Additionally, although the control and treatment groups did not have identical sample sizes, there was no difference in the demographics, implants, surgical approach and surgeon between the two groups. Thirdly, the hips in the study were not at a high risk for HO, since none of them had any of the well-identified risk criteria for developing HO postoperatively [7,9,10,11]. Therefore, it is not clear if these findings would be applicable to high-risk patients. However, the hips in this study typify the most common patient population undergoing a primary cementless THA. In addition, it is not clear from this study if the use of Celecoxib for a period of time longer than 10 days, but less than 6 weeks, would be more effective. However, previous studies at 6 weeks of treatment, compared to controls, did not demonstrate HO rates different than those of this study. Lastly, the Brooker classification system has its limitations in terms of reliability and reproducibility [69,70]. This variability was minimized by blinding the observer to the patient’s group and using both the AP pelvis and hip radiographs to determine the extent of HO.

## 5. Conclusions

A short 10-day course of Celecoxib significantly reduces the incidence of HO following cementless THA. This provides a prophylactic treatment option that is simple and minimizes the risk of complications associated with previous regimens using non-selective NSAIDs. In addition, Celecoxib provides the added benefit, after hip arthroplasty, of minimizing the need for narcotics and not interfering with postoperative anticoagulation prophylaxis.

## Figures and Tables

**Table 1:** Patient diagnoses.

		All Hips (n = 312)	Control Group (n = 104)	Celecoxib Group (n = 208)	*p*-Value
Diagnosis number (%)	OA	267 (85.6%)	86 (82.6%)	181 (87.0%)	0.58
	DDH	22 (7.1%)	9 (8.7%)	13 (6.3%)	
	AVN	23 (7.3%)	9 (8.7%)	14 (6.7%)	

OA: osteoarthritis; DDH: developmental dysplasia of the hip; AVN: avascular necrosis.

**Table 2:** Patient clinical characteristics.

		All Hips (n = 312)	Control Group (n = 104)	Celecoxib Group (n = 208)	*p*-Value
Mean Age (years)		64.2	64.3	64.2	0.46
Sex number (%)	Male	173 (55.4%)	63 (60.6%)	110 (52.9%)	0.19
	Female	139 (44.6%)	41 (39.4%)	98 (47.1%)	

**Table 3:** Heterotopic ossification and Brooker classification.

Brooker Class	All Hips (n = 312)	Control Group (n = 104)	Celecoxib Group (n = 208)	*p*-Value
I	50 (16.0%)	26 (25.0%)	24 (11.5%)	0.002
II	12 (3.8%)	3 (2.9%)	9 (4.3%)	0.53
III	10 (3.2%)	4 (3.8%)	6 (2.9%)	0.64
IV	0 (0.0%)	0 (0.0%)	0 (0.0%)	–
Total	72, (23.0%)	33 (31.7%)	39 (18.7%)	0.01

Results are presented as n = number of hips (%).

**Table 4:** Patient-reported outcome measures and range of motion.

		All Hips (n = 312)	Control Group (n = 104)	Celecoxib Group (n = 208)	*p*-Value
Mean PROM Score	WOMAC Stiffness	0.23	0.35	0.17	0.02
	WOMAC Physical Function	2.30	3.26	1.83	0.03
	WOMAC Pain	0.65	0.88	0.53	0.07
	HHS	95	95	96	0.24
	UCLA Activity Score	5.92	6.01	5.87	0.24
	SF-12 PCS	52	53	52	0.07
	SF-12 MCS	55	55	55	0.22

Results are presented as the mean value. PROM: patient-reported outcome measure; ROM: range of motion; WOMAC: Western Ontario and McMaster Universities Osteoarthritis Index; HHS: Harris Hip Score; UCLA: University of California, Los Angeles; SF-12: 12-Item Short Form Health Survey; PCS: physical component score; MCS: mental component score.

**Table 5:** Patient Range of Motion.

		All Hips (n = 312)	Control Group (n = 104)	Celecoxib Group (n = 208)	*p*-Value
ROM in degrees	Flexion	125	126	125	0.19
	Abduction	45	44	45	0.25
	Adduction	45	44	44	0.47
	Internal Rotation	44	44	44	0.21
	External Rotation	44	44	44	0.28

Results are presented as the mean value.
